# Standardized protocol for determination of biohydrogen potential

**DOI:** 10.1016/j.mex.2019.11.027

**Published:** 2019-12-04

**Authors:** Julián Carrillo-Reyes, Germán Buitrón, Iván Moreno-Andrade, Aida Cecilia Tapia-Rodríguez, Rodolfo Palomo-Briones, Elías Razo-Flores, Oscar Aguilar-Juárez, Jorge Arreola-Vargas, Nicolas Bernet, Adriana Ferreira Maluf Braga, Lucia Braga, Elena Castelló, Lucile Chatellard, Claudia Etchebehere, Laura Fuentes, Elizabeth León-Becerril, Hugo Oscar Méndez-Acosta, Gonzalo Ruiz-Filippi, Estela Tapia-Venegas, Eric Trably, Jorge Wenzel, Marcelo Zaiat

**Affiliations:** a Laboratory for Research on Advanced Processes for Water Treatment, Instituto de Ingeniería, Unidad Académica Juriquilla, Universidad Nacional Autónoma de México, Blvd. Juriquilla 3001, Queretaro, 76230, Mexico; b División de Ciencias Ambientales, Instituto Potosino de Investigación Científica y Tecnológica A.C., Camino a la Presa San José No. 2055, Col. Lomas 4a Sección, C.P. 78216, San Luis Potosí, SLP, Mexico; c Department of Environmental Technology, Centro de Investigación y Asistencia en Tecnología y Diseño del Estado de Jalisco, A.C., Av. Normalistas 800, Col. Colinas de la Normal, C.P. 44270, Guadalajara, Jalisco, Mexico; d División de Procesos Industriales, Universidad Tecnológica de Jalisco, Luis J. Jiménez No. 577, 1o de Mayo, C.P. 44979, Guadalajara, Jalisco, Mexico; e INRAE, Univ. Montpellier, LBE, Narbonne, France; f Biological Process Laboratory, São Carlos School of Engineering, University of São Paulo (LPB/EESC/USP), Av. João Dagnone 1100, São Carlos, São Paulo, 13563-120, Brazil; g Laboratorio BioProA, Facultad de Ingeniería, Universidad de la República de Uruguay, Av. Julio Herrera y Reissig 565, Montevideo, Uruguay; h Laboratorio de Ecología Microbiana, Departamento de Bioquímica y Genómica Microbiana, Instituto de Investigaciones Biológicas Clemente Estable, Av. Italia 3318, Montevideo, Uruguay; i Departamento de Ingeniería Química, CUCEI-Universidad de Guadalajara, Blvd. M. García Barragan 1451, C.P. 44430, Guadalajara, Jalisco, Mexico; j Escuela de Ingeniería Bioquímica, Facultad de Ingeniería, Pontificia Universidad Católica de Valparaíso, Av. Brasil 2085, Valparaíso, Chile

**Keywords:** Automatic protocol, Dark fermentation, Heat-treated inoculum, Manual protocol

## Abstract

Biohydrogen production potential (BHP) depends on several factors like inoculum source, substrate, pH, among many others. Batch assays are the most common strategy to evaluate such parameters, where the comparison is a challenging task due to the different procedures used. The present method introduces the first internationally validated protocol, evaluated by 8 independent laboratories from 5 different countries, to assess the biohydrogen potential. As quality criteria, a coefficient of variation of the cumulative hydrogen production (*H*_max_) was defined to be <15 %. Two options to run BHP batch tests were proposed; a manual protocol with periodic measurements of biogas production, needing conventional laboratory materials and analytical equipment for biogas characterization; and an automatic protocol, which is run in a device developed for online measurements of low biogas production. The detailed procedures for both protocol options are presented, as well as data validating them. The validation showed acceptable repeatability and reproducibility, measured as intra- and inter-laboratory coefficient of variation, which can be reduced up to 9 %.

## Graphical abstract

**Figure:**
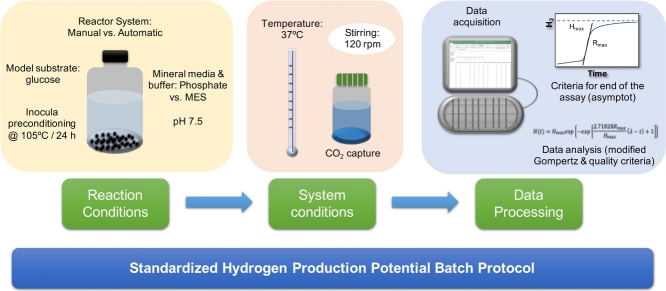

**Specification Table**

**Table:** 

Subject Area:	Environmental Science
More specific subject area:	*Biofuels production*
Protocol name:	*Biohydrogen potential protocol*
Reagents/tools:	*Required reagents*
	• NH_4_Cl • MES (2-(N-Morpholino) ethanesulfonic acid, 4-Morpholineethanesulfonic acid) • MgCl_2_∙6H_2_O • FeSO_4_∙7H_2_O • CoCl_2_∙6H_2_O • MnCl_2_∙4H_2_O • KI • NiCl_2_∙6H_2_O • ZnCl_2_ • Anhydrous glucose • HCl (37 %) • NaOH • Thymolphthalein (only for the automatic version) • Distilled water
	*Required materials and equipment*
	• 10-mL graduated pipettes or an air displacement pipette (1−10 mL) with tips • Spatula • pH meter • Pasteur pipettes • Digital analytical balance (readability down to 0.1 mg) • Industrial blender, electric coffee grinder or porcelain mortar and pestle • Standard mesh # 20 (sieve size 850 μm) • Laboratory oven at 105 ± 1 °C • Gas chromatograph to analyze biogas composition (H_2_ and CO_2_)
	Specific materials for the manual protocol
	• 120-mL serum bottles (Fig. 1a) **Fig. 1:** 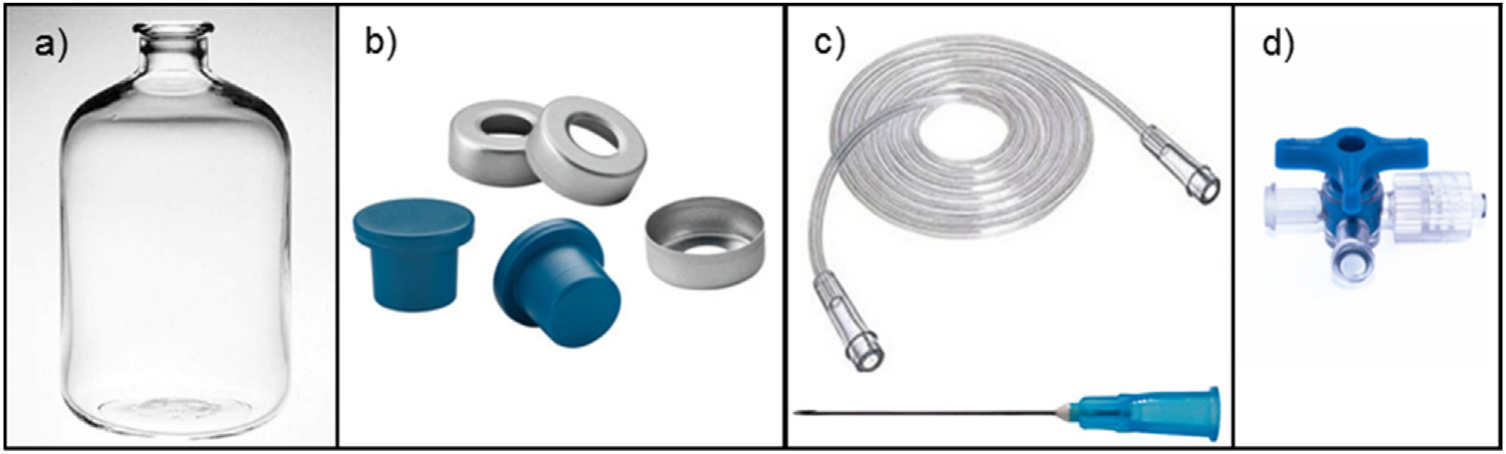 Serum bottles, rubber stoppers, and aluminum rings. • Rubber stoppers for serum bottles (for anaerobic cultures) (Fig. 1b) (Note: Be careful to not use thin stoppers that can lead to leakage of the gas after the first puncture of the needle) • Aluminum rings (Fig. 1b) • 18-G x 1 1/2-in hypodermic needles and tubing (Fig. 1c) • Medical disposable three-way stopcock valve (Fig. 1d) • 50-mL plastic syringe • 100-mL graduated cylinder • 500-mL glass beaker • Crimper to seal aluminum rings • Incubator with orbital agitation • Glass thermometer
	Specific materials for the automatic protocol
	• Automatic Methane Potential Test System (AMPTS II, Bioprocess Control AB; Lund Sweden) (Fig. 2) **Fig. 2:** 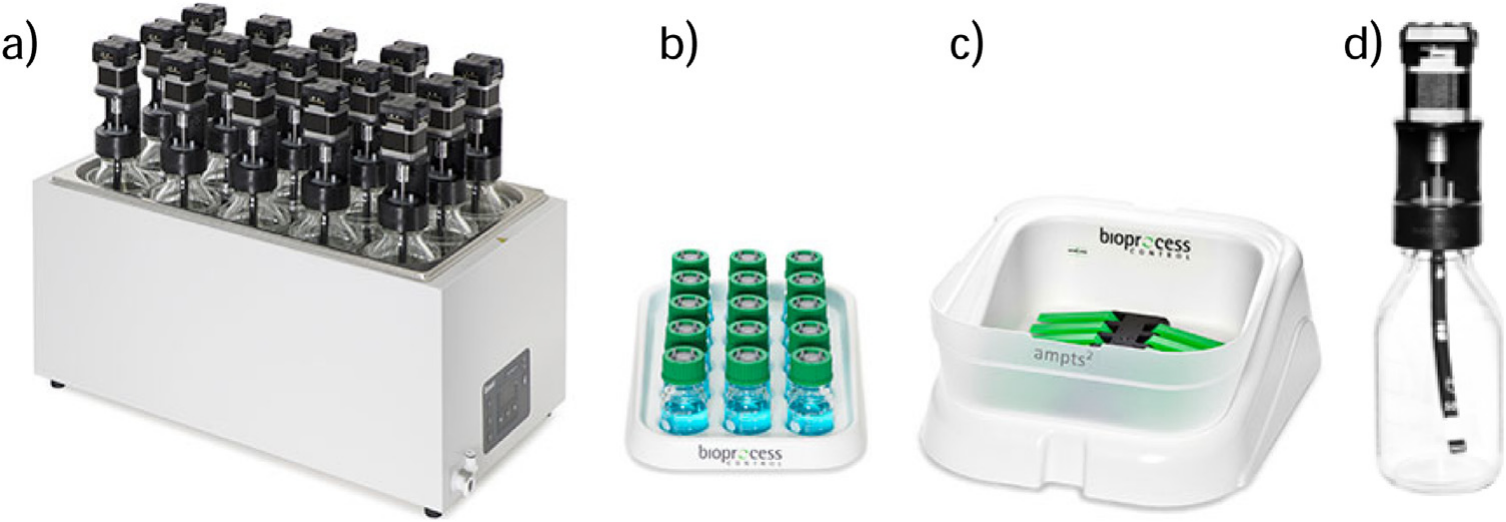 Sample incubation unit (a), CO_2_ absorption unit (b), gas volume measuring device (c), and Schott bottles with agitation system (d). • 500-mL Schott glass bottles (Fig. 2d) • 50-mL graduated cylinder • 500-mL graduated cylinder • 50-mL glass beakers
Experimental design:	*The proposed method presents two options to run BHP batch tests; a manual protocol with periodic measurements of biogas production; and an automatic protocol, which is run in a device developed for online measurements of low biogas production.*
Trial registration:	*n/a*
Ethics:	*n/a*

**Value of the Protocol**

**Table:** 

• *This method stands for the first standardized protocol validated internationally to estimate the hydrogen potential.* • *Two versions of the protocol are included, a manual one with low equipment’s requirements and an automatic one.* • *The average intra-laboratory coefficient of variation can be reduced up to* 9 %.

## Description of protocol

### Background

Hydrogen production by dark fermentation is an emerging technology of increasing interest due to its renewable feature. The assessment of the BHP is a common procedure to evaluate new potential substrates, and other parameters [[1], [2], [3]]. This method stands for the first standardized protocol validated internationally to estimate the BHP using batch tests. A complete discussion of the development of this protocol has been published [4].

### Stock solutions

Solution A: nutrients and buffer solution, modified from [5]

Dissolve in a volumetric flask to make 1 L solution with distilled water: 41.6 g NH_4_Cl, 19.52 g MES, 2 g MgCl_2_∙6H_2_O, 1.6 g FeSO_4_∙7H_2_O, 40 mg CoCl_2_∙6H_2_O, 40 mg MnCl_2_∙4H_2_O, 40 mg KI, 8 mg NiCl_2_∙6H_2_O, 8 mg ZnCl_2_.

Solution B: substrate, 50 g L^−1^ glucose

Dry 28 g of anhydrous glucose (105 °C, 2 h) and allow to cool in a desiccator. Weigh 25 g of dry glucose and dissolve with distilled water to make a 500-mL solution in a volumetric flask. The source of carbohydrates can vary according to the aim of each determination, e.g., using an agroindustrial effluent. The recommendation to get the maximum hydrogen potential is to keep the initial carbohydrate concentration at 5 g L^−1^ during the assay.

Solution C: pH adjustment, 5 N HCl

Add 25 mL of distilled water to a 50-mL volumetric flask. Add 20 mL of HCl (37 %) and dilute to the graduation marking with distilled water.

Solution D: pH adjustment, 5 N NaOH

Add 10 g of NaOH to 30 mL of distilled water, dissolve with magnetic stirring and allow to cool. Dilute the solution with distilled water to complete 50 mL in a volumetric flask.

Solution E: pH indicator, 0.4 % (w/v) thymolphthalein

Dissolve 40 mg of thymolphthalein in 9 mL of ethanol (99.5 %); dilute the solution with distilled water to complete 10 mL in a volumetric flask.

Solution F: CO_2_ absorption solution, 3 N NaOH

Add 144 g of NaOH to 1 L of distilled water, dissolve with agitation; dilute it to 1.2 L. Add 6 mL of solution E.

Note: Solutions E and F are only required for the automatic protocol.

### Inoculum preparation

Use sludge from an anaerobic wastewater treatment reactor as inoculum. Perform a thermal pretreatment drying the sludge at 105 °C during 24 h, for the selection of hydrogen-producing microorganisms [6]. Let the sludge cool and homogenize it by grinding (with the blender, coffee grinder or the mortar) and select the particle size lesser than 850 μm by sieving. Determine volatile solids (VS) content according to standard methods [7].

### Manual protocol

Preparation

**Fig. 3:**
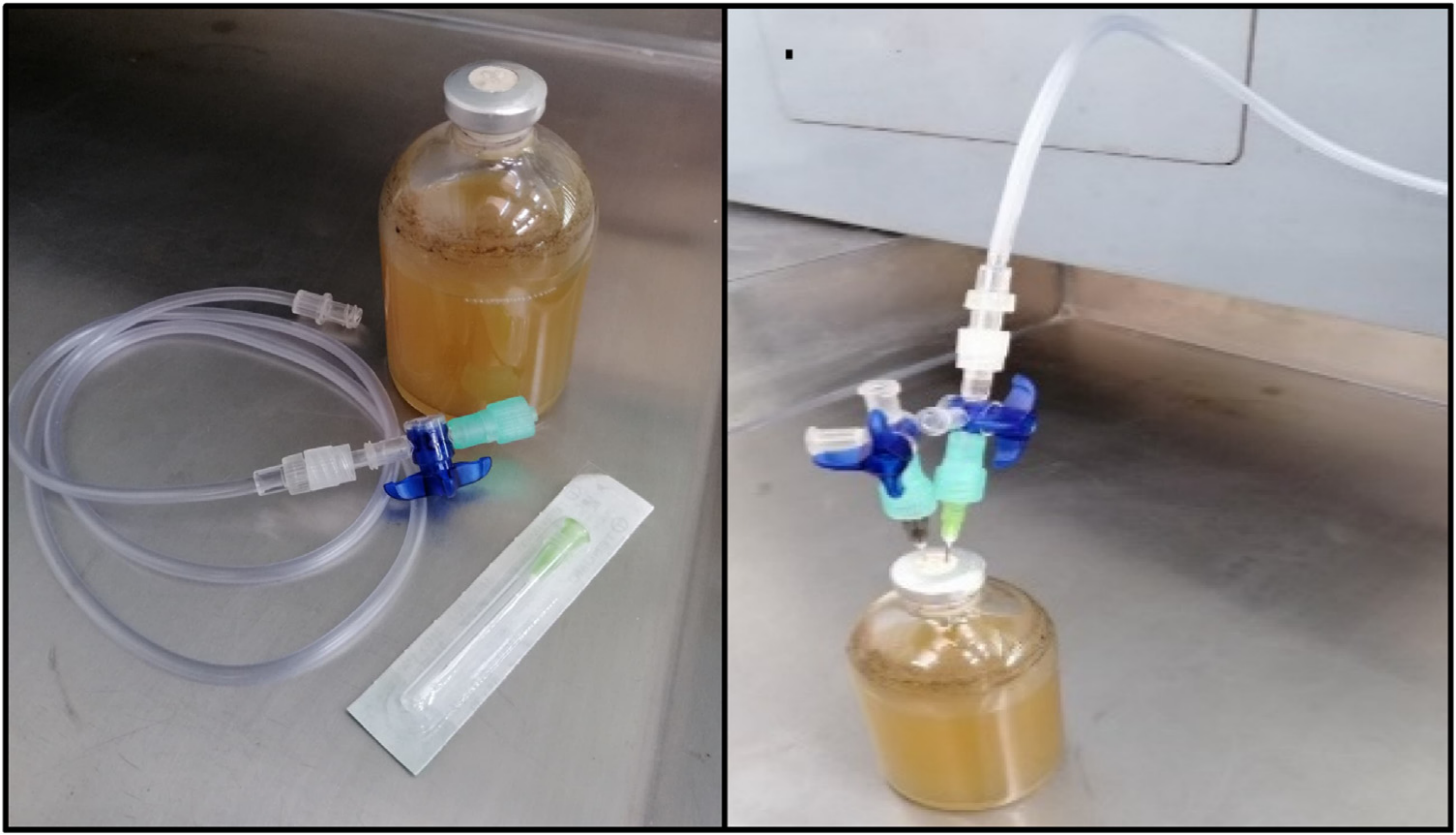
Headspace exchange on the serum bottles.

1 Label all bottles; triplicates of each evaluated condition must be included, as well as endogenous control (without substrate) in triplicate.
2 Calculate the necessary amount of inoculum to add to each serum bottle to maintain an S/X ratio (substrate/inoculum, g substrate g VS^−1^) of 2.7, considering a working volume of 80 mL and an initial carbohydrate concentration of 5 g L^−1^. Weigh the exact amount of inoculum directly in the serum bottle using the analytical balance.
3 Add 5 mL of solution A in each bottle using a graduated pipette (or an air displacement pipette).
4 Add 4 mL of solution B (with the exception of the endogenous control) using a graduated pipette (or an air displacement pipette); the initial glucose concentration will be 5 g L^−1^.
5 Add the needed amount of distilled water using a graduated cylinder, 71 mL or 75 mL, for bottles with carbohydrates (with solution B) or endogenous controls, respectively; to complete 80 mL of working volume in the serum bottles. The headspace will be of 40 mL.
6 Adjust the initial pH to 7.5 by dripping solution C or D as necessary using a Pasteur pipette.
7 Close the bottles with the rubber stopper and seal them with the aluminum rings (use the crimper).
8 For the headspace exchange, insert a needle connected to a hose to flush with N_2_ for 30 s in the serum bottles and with the help of another needle to let the gas out (Fig. 3). After 30 s, get out the needles simultaneously.

Incubation and monitoring of H_2_ production

**Fig. 4:**
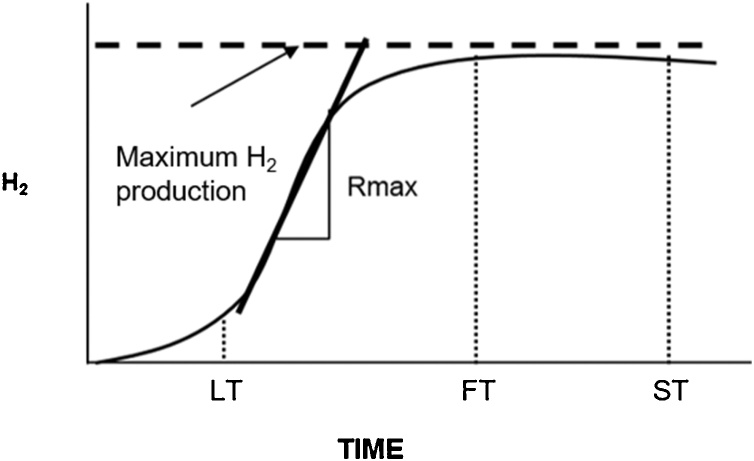
Kinetic over time. LT: latency time; FT: final time; SP: time to stop the kinetic.
7 To stop the test, remove the serum bottles from the incubator. See the sampling and analysis section for further instructions.

NOTE. To determine the end of the test properly, you will have to plot immediately every H_2_ production reading.

1 Considering previous experimental experiences to validate this protocol, start the incubation close to noon (12:00 h).
2 Incubate the bottles at 37 °C with an orbital shaking of 150 rpm. After this step, prepare the water-filled inverted graduated cylinder according to the following section.
3 After one hour of incubation, discharge the excess pressure of each serum bottles in the inverted graduated cylinder.
4 Perform gas production measurements every three or four hours. Record the volume produced, as well as the temperature of the acid water in the water-filled inverted graduated cylinder.
5 To take a gas sample, purge the hose before taking the sample. Analyze the composition of the biogas (H_2_ and CO_2_) at least once a day by gas chromatography.
6 To determine the end of the test, apply the stop time criterion (ST), which is obtained when the H_2_ production curve becomes asymptotic (FT) (Fig. 4) [8]. Additionally, you can use the coefficient of variation between the last three records of cumulative hydrogen as stop criterion when it is lower than 5 %.

Water-filled-inverted graduated cylinder to measure biogas

**Fig. 5:**
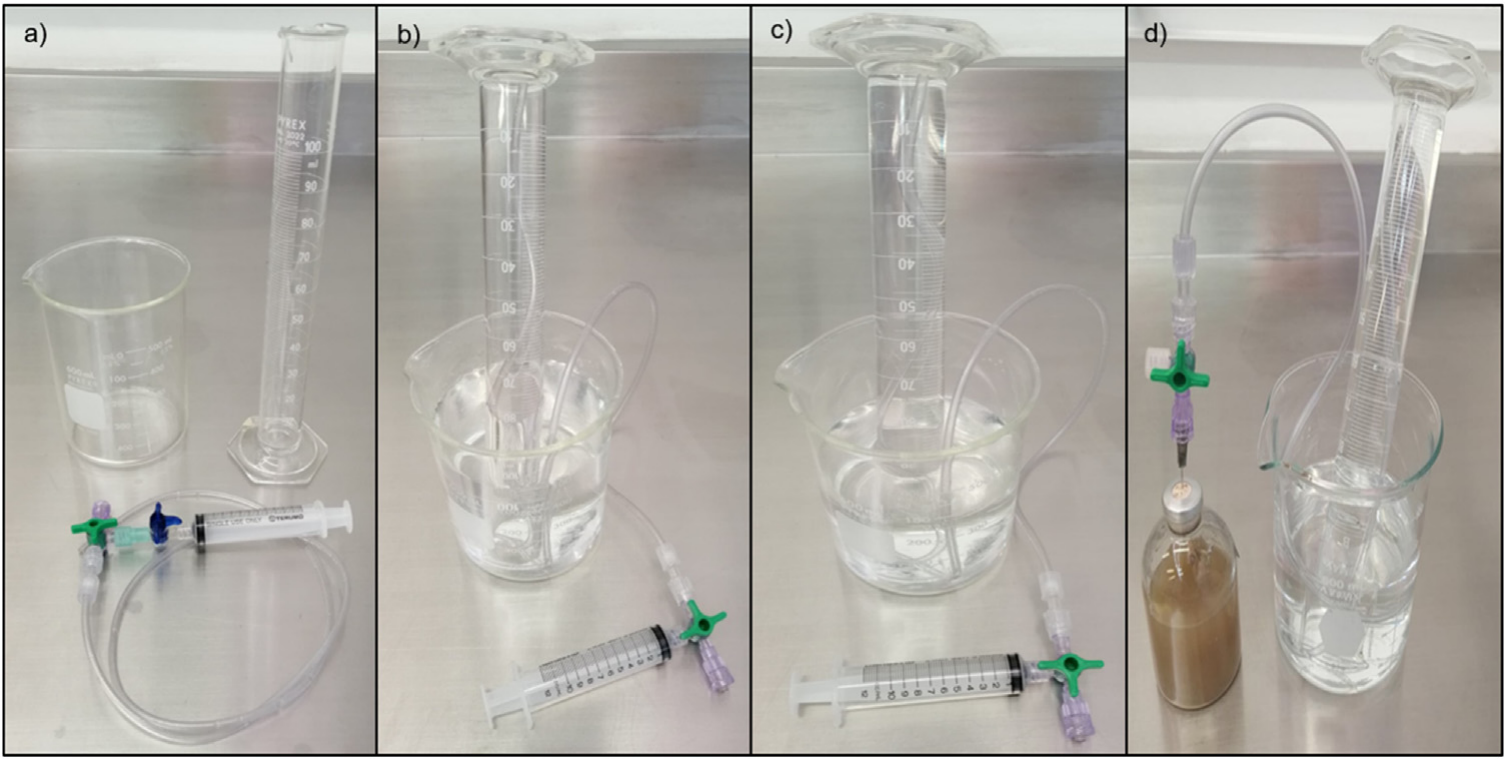
Required material for the biogas measuring (a), filling the inverted cylinder with a syringe (b), water-filled-inverted graduated cylinder (c), and biogas production measuring (d).
3 To measure the biogas production, connect a needle to one side of the three-way stopcock and keep it closed, then insert the needle through the rubber stopper of the serum bottles and open the stopcock to allow the biogas displace the liquid in the cylinder, register the volume (Fig. 5d).
4 The biogas samples can be taken from the displaced volume in the cylinder, or using a chromatography syringe directly from the serum bottle.

1 Acidify 500 mL of distilled water with 10 mL of solution C (pH < 2) to prevent CO_2_ dissolution in the liquid.
2 Place a 100 mL inverted cylinder as shown in Fig. 5 using a hose to the bottom of the cylinder, a syringe, and a three-way stopcock to fill the cylinder with the acid water (Fig. 5c).

### Automatic protocol

For the automatic determination of the biohydrogen potential, the protocol was adapted to the manufacturer recommendations of the Automatic Methane Potential Test System II (Bioprocess Control AB; Lund, Sweden), in the operation and maintenance manual [https://www.bioprocesscontrol.com/media/1511/bioprocess-control-manual-ampts-ii-ampts-ii-light.pdf].

### Preparation

1 For the CO_2_ absorption unit preparation, add 80 mL of F solution to each bottle of the CO_2_ absorption unit (Fig. 3b), place a plastic stopper with the two tubing ports.
2 Connect the biogas hoses of each bottle of the CO_2_ absorption unit to the correspondent bottle in the incubation unit (Fig. 3a) according to the AMPTS II instructions.
3 Label all Schott bottles; triplicates of each evaluated condition must be included, as well as endogenous control (or blank) in triplicate.
4 Calculate the necessary amount of inoculum to add to each Schott bottle to maintain an S/X ratio (substrate/inoculum, g substrate g VS^−1^) of 2.7, considering a working volume of 360 mL and an initial carbohydrate concentration of 5 g L^−1^. Weigh the exact amount of inoculum for each bottle in glass beakers using the analytical balance.
5 Add 22.6 mL of solution A in each glass bottle using a graduated pipette (or an air-displacement pipette).
6 Add 36 mL of solution B in each bottle (except to endogenous control) using a graduated pipette (or an air displacement pipette); the initial glucose concentration will be 5 g L^−1^.
7 Add the weighed inoculum to each glass bottle, suspend the remaining sludge that stays in the glass beaker using the water pointed out in the following step, and added to the corresponding bottle.
8 To complete 360 mL of working volume in the serum bottles, add the needed amount of distilled water using a graduated cylinder and pipette, 301.4 and 337.4 mL, to the bottles with carbohydrates (with solution B) or for endogenous controls, respectively. The headspace will be of 240 mL. A working volume/headspace ratio of 1.5 will be maintained according to the recommendations of the AMPTS II manual.
9 Adjust the initial pH to 7.5 by dripping solution C or D as necessary using a Pasteur pipette.
10 Put a thin layer of stopcock grease in the bottle thread and the outside part of the bottle opening, to avoid gas leaks.
11 Place the bottle stoppers with two tubing ports and rotating shaft for mixing in the opening of the bottles.
12 Attach the multifunction brushless DC motor.
13 Put the Tygon® hose in the ports from the stopper. Connect one of the hoses to the corresponding bottle in the CO_2_ absorption unit and seal the other hose with a pipe plug.

Headspace exchange

**Fig. 6:**
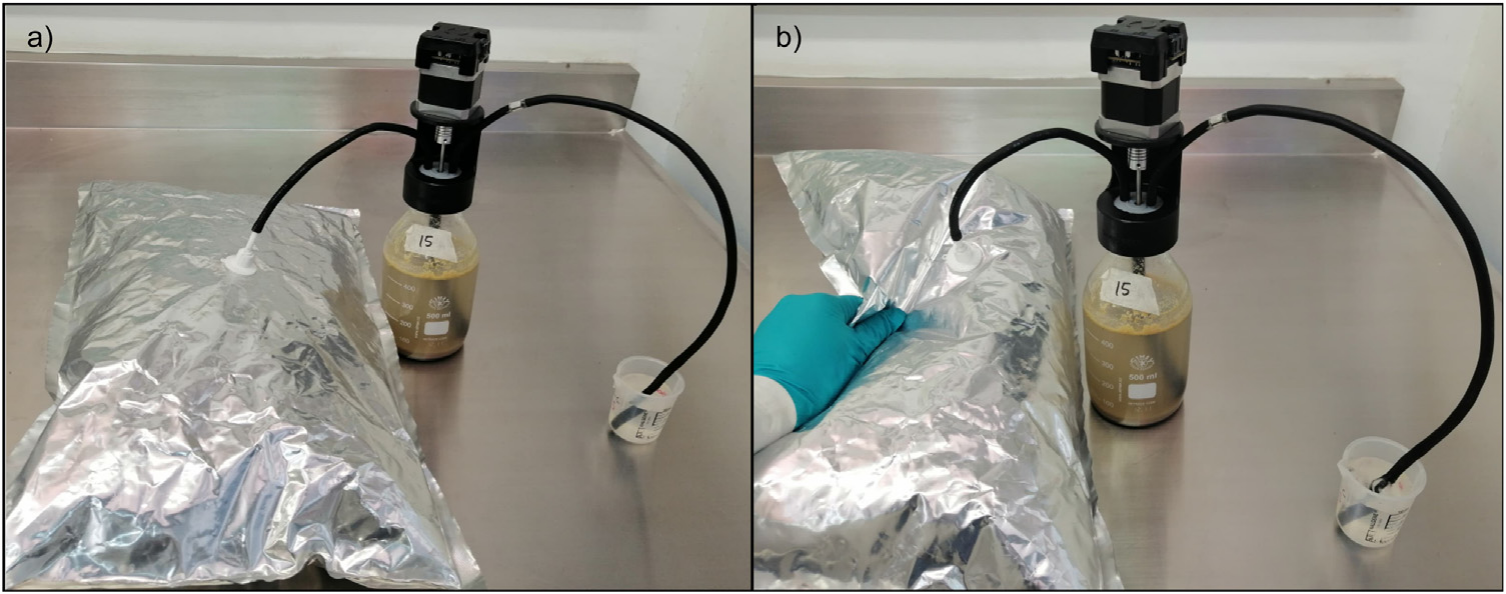
Headspace exchange on AMPTS II bottles using a gas sampling bag with N_2_.
2 Close the Tygon® hoses again with the pipe plug and reconnect the other hose to the corresponding bottle in the CO_2_ absorption unit.

1 For the headspace exchange, temporally disconnect the Tygon® hose from the CO_2_ absorption unit and introduce the hose in a beaker with water to discharge the excess pressure. Remove the pipe plug and connect it to a N_2_ flow, flush the headspace during 30 s (Fig. 6).

AMPTS II system incubation and monitoring

1 Fill the incubation unit with distilled water and set to a temperature of 37 °C.
2 Connect the agitation systems of each bottle serially with the motor cables, and the first motor to the Motor Controller.
3 Connect the 12 V power adapter to the Gas Volume Measuring Device, and to a 100−240 V 50/60 Hz standard power socket. After this, connect the 24 V power adapter to the Motor Controller and to a 100−240 V 50/60 Hz standard power socket.
4 Set the System switch on the Motor Controller to ON, and set the ON/OFF switches on each motor unit to ON.
5 Empty each flow cell in the gas volume measuring device.
6 Adjust the motor speed to 60 % (approx. 120 rpm), with intermittent mixing at 60 s ON and 180 s OFF. Activate the motor setting the control to ON and apply the settings.
7 Start the data logging program according to the AMPTS II software manual.
8 Analyze the gas composition to determine the hydrogen fraction in the biogas samples, for each bottle at least once during the kinetics. Disconnect the Tygon® hose from the absorber unit and seal it with a pipe plug and take the biogas sample with a syringe from the other hose.
9 Check daily that the hard tubing pieces that connect the bent stir rod to the motor are undamaged and the mixing works properly. Otherwise, change the tubing pieces.
10 Check daily that the water level in the thermostatic water bath reaches the plastic lid and fill it with additional distilled water if necessary. Make sure that the Tygon® hose is not bent abruptly, interfering with the gas flow and not being damaged by the rotating parts of the agitator/motor.
11 Verify that the water level in the gas volume measurement device is within the recommended range. Fill with additional deionized or distilled water to the recommended water level when necessary.
12 The thymolphthalein pH indicator in solution F will change from blue to colorless when the CO_2_ absorption capacity of the NaOH solution drops below the optimum. At this point, it is recommended to replace the bottle with new solution F to prevent the CO_2_ gas from passing to the gas volume measuring device. Approximately 2.9 L of CO_2_ can be captured (at 22 °C) in each bottle before it needs to be changed.
13 To change the solution F, place pipe plugs on the hoses of the incubation unit and gas measuring device. Replace the old solution with a new one, reconnect the hoses and remove the pipe plugs.

NOTE. Before using the AMPTS II system, read the operation and maintenance manual carefully, details of setting up the equipment, connection, and software management are included.

End of the test

1 The AMPTS II software automatically upgrades the cumulative hydrogen production curve; review the curve daily to determine the end of the essay following the stop criteria. The stop time criterion (ST) is obtained when the H_2_ production curve becomes asymptotic (FT) (Fig. 4) [8]. As an additional stop criterion, you can use the coefficient of variation between the last three records of cumulative hydrogen when it becomes lower than 5 %.
2 In the AMPTS II software, generate a report in the Download report menu. Download the report and open it to make sure that the report has been generated correctly and that no errors have occurred during the download of the file.
3 Stop the registration by pressing the pause button and then the stop button. Note: By pressing the stop button, the experiment associated with that particular cell can no longer continue.
4 Turn off the thermostatic water bath.
5 Set the on/off switch of each motor unit to OFF.
6 Set the system switch on the motor controller to OFF.
7 Unplug the power adapters (for the motor controller and the gas volume measuring device) from the power supply.
8 Disconnect the hoses between the reactors and the NaOH bottles.
9 Disconnect the hoses between the NaOH bottles and the gas volume measuring device.
10 Empty the water bath using the manual water pump included with the equipment.

Sampling and analysis

1 Once the kinetics have been completed according to the previously mentioned criteria, open the bottles and record the pH value of its liquid content.
2 Keep the bottles in constant agitation with the help of a magnetic stirrer and grill. Take a homogeneous sample of at least 20 mL from each bottle and determine the concentration of total and volatile suspended solids by duplicate (5 mL per sample) according to standard methods [7].
3 Take a sample of at least 20 mL from each bottle to determine the chemical oxygen demand (COD), fermentation products, and residual carbohydrates. Acidify the samples with concentrated H_2_SO_4_ (pH < 2).
4 Centrifuge the 20-mL samples (3600 rpm for 10 min, or at a higher speed) and filter the supernatant with 0.45 μm nitrocellulose filters. The sample can be kept refrigerated (4 °C) for no more than 7 days until analysis. If samples contain many suspended solids, before the nitrocellulose filter it is advisable to use a 1.5 μm glass fiber filter.
5 From the centrifuged sample analyze the COD according to standard methods [7], the fermentation products by gas or liquid chromatography, as well as the residual carbohydrates by the phenol-sulfuric acid method [9].

Validation of analytical methods

It is highly recommended to validate the analytical methods to determine COD, sugars and fermentation products as indicated below:

1 Weigh 1 g of anhydrous glucose (dried at 105 °C for 2 h and stored in a desiccator) and dilute in a 1 L volumetric flask with distilled water.
2 Analyze the COD and carbohydrates of the solution with the methodology of each laboratory. Consider that glucose has a theoretical COD of 1.067 g COD g glucose^−1^.
3 If the results of COD or carbohydrates analysis are not as expected, you should review your analysis procedure and calibration curves.
4 To validate the methods of measuring fermentation products it is suggested to use certified reference materials (e.g. Volatile Free Acid Mix CRM46975-Supelco, and Lactic acid PHR1215-Supelco; Merck KGaA, Germany).

### Calculations

#### H_2_ cumulative volume

From the cumulative hydrogen volume graph adjust the curve to the modified Gompertz model [10], record the software used and the correlation coefficient, make the adjustment for each replicate:

$$H(t)=H_{\text{m}\text{a}\text{x}}exp\left\{-exp\left[\frac{2.71828R_{\text{m}\text{a}\text{x}}}{H_{\text{m}\text{a}\text{x}}}\left(\lambda -t\right)+1\right]\right\}$$

Where *H(t)* (mL) is the cumulative hydrogen production at time t (h); *H*_max_ (mL) is the maximum gas produced; *R*_max_ is the maximum production rate (mL h^−1^); and *λ* is the lag-phase time before the exponential hydrogen production (h). For cumulative hydrogen production results using the manual version, adjust the recorded volume to standard conditions (0 °C and 1 atm). The automatic device AMPTS II reports the gas production at standard conditions, according to internal temperature and pressure sensors.

#### COD balance of fermentation products

It is highly recommended to quantify the non-identified (N.I._CODeq_) products following a COD balance [11]:

$${N.I.}_{CODeq=}{COD}_{f}-{Glucose}_{CODeq}-\sum {Metabolites}_{CODeq}$$

Where ${COD}_{f}$ (g_COD_ L^−1^) is the analytical soluble COD concentration determined at the end of the test; ${Glucose}_{CODeq}$ (g_CODeq_ L^−1^) is the residual glucose concentration; and $\sum {Metabolites}_{CODeq}$ is the total soluble products concentration (g_CODeq_ L^−1^) determined by chromatography. The COD equivalence of the different fermentation products and carbohydrates, can be calculated with the following equations, based on the average oxidation reactions of the analyzed compounds and the water-oxygen as electron acceptor.

$$a)\;\frac{{mg\;COD}_{ace}}{L}=\left|\frac{{X\;mg}_{ace}}{L}\right|\left|\frac{1\;mmol}{{60\;mg}_{ace}}\right|\left|\frac{8\;meq\;e^{-}}{{1\;mmol}_{ace}}\right|\left|\frac{8\;mg\;OD}{1\;meq\;e^{-}}\right|$$

$$b)\;\frac{{mg\;COD}_{pro}}{L}=\left|\frac{{X\;mg}_{pro}}{L}\right|\left|\frac{1\;mmol}{{74\;mg}_{pro}}\right|\left|\frac{14\;meq\;e^{-}}{{1\;mmol}_{pro}}\right|\left|\frac{8\;mg\;OD}{1\;meq\;e^{-}}\right|$$

$$c)\;\frac{{mg\;COD}_{but}}{L}=\left|\frac{{X\;mg}_{but}}{L}\right|\left|\frac{1\;mmol}{{88\;mg}_{but}}\right|\left|\frac{20\;meq\;e^{-}}{{1\;mmol}_{but}}\right|\left|\frac{8\;mg\;OD}{1\;meq\;e^{-}}\right|$$

$$d)\;\frac{{mg\;COD}_{val}}{L}=\left|\frac{{X\;mg}_{val}}{L}\right|\left|\frac{1\;mmol}{102{\;mg}_{val}}\right|\left|\frac{12\;meq\;e^{-}}{{1\;mmol}_{val}}\right|\left|\frac{8\;mg\;OD}{1\;meq\;e^{-}}\right|$$

$$e)\;\frac{{mg\;COD}_{cap}}{L}=\left|\frac{{X\;mg}_{cap}}{L}\right|\left|\frac{1\;mmol}{{116\;mg}_{cap}}\right|\left|\frac{24\;meq\;e^{-}}{{1\;mmol}_{cap}}\right|\left|\frac{8\;mg\;OD}{1\;meq\;e^{-}}\right|$$

$$f)\;\frac{{mg\;COD}_{eth}}{L}=\left|\frac{{X\;mg}_{eth}}{L}\right|\left|\frac{1\;mmol}{{46\;mg}_{eth}}\right|\left|\frac{12\;meq\;e^{-}}{{1\;mmol}_{eth}}\right|\left|\frac{8\;mg\;OD}{1\;meq\;e^{-}}\right|$$

$$g)\;\frac{{mg\;COD}_{lac}}{L}=\left|\frac{{X\;mg}_{lac}}{L}\right|\left|\frac{1\;mmol}{{90\;mg}_{lac}}\right|\left|\frac{12\;meq\;e^{-}}{{1\;mmol}_{lac}}\right|\left|\frac{8\;mg\;OD}{1\;meq\;e^{-}}\right|$$

$$h)\;\frac{{mg\;COD}_{for}}{L}=\left|\frac{{X\;mg}_{for}}{L}\right|\left|\frac{1\;mmol}{{46\;mg}_{for}}\right|\left|\frac{2\;meq\;e^{-}}{{1\;mmol}_{for}}\right|\left|\frac{8\;mg\;OD}{1\;meq\;e^{-}}\right|$$

$$i)\;\frac{{mg\;COD}_{suc}}{L}=\left|\frac{{X\;mg}_{suc}}{L}\right|\left|\frac{1\;mmol}{{118\;mg}_{suc}}\right|\left|\frac{14\;meq\;e^{-}}{{1\;mmol}_{suc}}\right|\left|\frac{8\;mg\;OD}{1\;meq\;e^{-}}\right|$$

$$j)\;\frac{{mg\;COD}_{glu}}{L}=\left|\frac{{X\;mg}_{glu}}{L}\right|\left|\frac{1\;mmol}{{180\;mg}_{glu}}\right|\left|\frac{24\;meq\;e^{-}}{{1\;mmol}_{glu}}\right|\left|\frac{8\;mg\;OD}{1\;meq\;e^{-}}\right|$$

$$k)\;{mg\;COD}_{H_{2}}=\left|{X\;mL\;}_{H2,\;STP}\right|\left|\frac{1\;mmol}{{22.4\;mL}_{H2}}\right|\left|\frac{2\;meq\;e^{-}}{{1\;mmol}_{H2}}\right|\left|\frac{8\;mg\;OD}{1\;meq\;e^{-}}\right|$$

Where $\frac{X{mg}_{compound}}{L}=$ produced metabolites concentration $\;\left(\frac{mg}{L}\right)$; *ace*, acetic acid; pro, propionic acid; *but*, butyric and isobutyric acids; *val*, valeric and isovaleric acids; *cap*, caproic and isocaproic acids; *eth*, ethanol; *lac*, lactic acid; *for*, formic acid; *suc*, succinic acid; *glu*, glucose; *H_2_*, hydrogen; *X mL _H2, STP_*, hydrogen volume at standard temperature (0 °C) and pressure (1 atm) conditions. The COD equivalence is also useful to determine the stoichiometry of the reactions and metabolic pathways followed by the inoculum used. An example of such calculation is detailed elsewhere [12].

### Quality criteria for the results

In order to validate the hydrogen production tests, the coefficient of variation of *H*_max_ must be <15 %, even after applying a statistical analysis to eliminate outliers [4]. Use the Dixon test to eliminate a unique outlier of triplicates, considering a *p* value = 0.05.

## Method validation

The present method was validated in 8 independent laboratories from 5 different countries (Brazil, Chile, France, Mexico and Uruguay); a detailed discussion about its development, validation and consideration of workload time were already published [4]. Average values of cumulative hydrogen production (*H*_max_) and the corresponding standard deviations were used to calculate the intra-laboratory and inter-laboratory coefficient of variation (CV) as repeatability and reproducibility indicators, respectively.

**Fig. 7:**
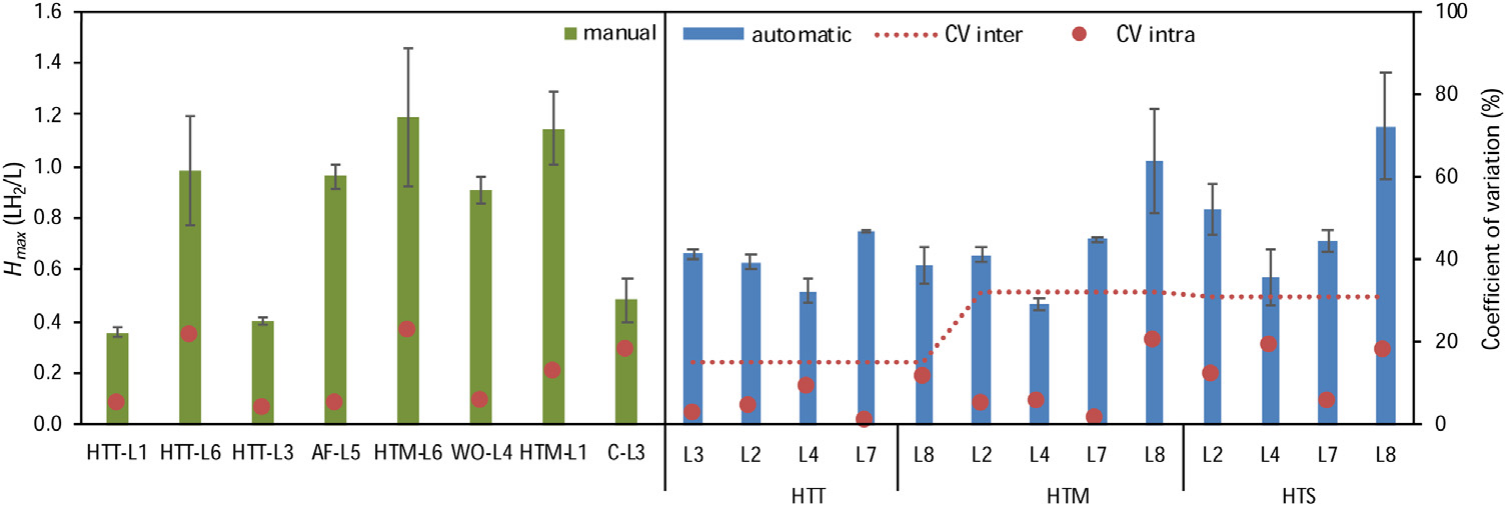
Cumulative hydrogen production (*H*_max_) specific to the working volume (L H_2_ L^−1^), for the manual and automatic protocols, and its corresponding intra- and inter-laboratory variation. For the manual protocol evaluation different inoculum were tested by different laboratories, meanwhile for the automatic protocol three different source of inoculum were evaluated in different laboratories. The different inoculum sources were heat-treated anaerobic sludge from a thermophilic digester (HTT); heat-treated anaerobic sludge from a mesophilic digester (HTM); biomass from an auto-fermented effluent rich in sucrose (AF); aerobic sludge pretreated by cell wash-out (WO); compost from kitchen wastes (C); and heat-treated anaerobic sludge from a digester treating solid wastes (HTS). L1–L8 stands for results from different laboratories.

Applying the present protocol, the average intra-laboratory coefficient of variation ranged from 13 % to 9 % using the manual and the automatic protocol, respectively. But using the automatic protocol, the interlaboratory variation could be reduced up to 15 % with the HTT as inoculum; which is an acceptable result according to studies analyzing the biochemical methane potential (Fig. 7) [13].

## Conclusions

This method features a new and validated protocol for biohydrogen potential, providing a tool for inter-laboratory studies and reliable comparisons of results. Indeed, following the protocol, the variation of results can be reduced to acceptable levels, using either the manual or automatic version.

## Declaration of Competing Interest

The authors declare that they have no known competing financial interests or personal relationships that could have appeared to influence the work reported in this paper.
